# HIV-1 Recruits UPF1 but Excludes UPF2 to Promote Nucleocytoplasmic Export of the Genomic RNA

**DOI:** 10.3390/biom5042808

**Published:** 2015-10-20

**Authors:** Lara Ajamian, Karen Abel, Shringar Rao, Kishanda Vyboh, Francisco García-de-Gracia, Ricardo Soto-Rifo, Andreas E. Kulozik, Niels H. Gehring, Andrew J. Mouland

**Affiliations:** 1 HIV-1 RNA Trafficking Laboratory, Lady Davis Institute for Medical Research-Sir Mortimer B. Davis Jewish General Hospital, Montréal QC H3T 1E2, Canada; E-Mails: lara.ajamian@mail.mcgill.ca (L.A.); karen_abel@gmx.de (K.A.); shringar.rao@mail.mcgill.ca (S.R.); kishanda.vyboh@mail.mcgill.ca (K.V.); 2 Department of Medicine, Division of Experimental Medicine, McGill University, Montréal QC H3A 2B4, Canada; 3 Department of Microbiology and Immunology, McGill University, Montréal QC H3T 1E2, Canada; 4 Laboratory of Molecular and Cellular Virology, Virology Program, Biomedical Sciences Institute, Faculty of Medicine, Universidad de Chile, Independencia 8389100, Santiago, Chile; E-Mails: franciscogarcia96@gmail.com (F.G.-G.); rsotorifo@med.uchile.cl (R.S.-R.); 5 Department of Pediatric Oncology, Hematology and Immunology, Heidelberg 69120, Germany; E-Mail: andreas.kulozik@med.uni-heidelberg.de; 6 European Molecular Biology Laboratory, Partnership Unit, University of Heidelberg Molecular Medicine, Heidelberg 69117, Germany; 7 Institute for Genetics, University of Cologne, Cologne 50674, Germany; E-Mail: ngehring@uni-koeln.de

**Keywords:** HIV-1, UPF1, ribonucleoprotein, nuclear RNA export, viral evasion, nonsense mediated decay

## Abstract

Unspliced, genomic HIV-1 RNA (vRNA) is a component of several ribonucleoprotein complexes (RNP) during the viral replication cycle. In earlier work, we demonstrated that the host upframeshift protein 1 (UPF1), a key factor in nonsense-mediated mRNA decay (NMD), colocalized and associated to the viral structural protein Gag during viral egress. In this work, we demonstrate a new function for UPF1 in the regulation of vRNA nuclear export. We establish that the nucleocytoplasmic shuttling of UPF1 is required for this function and demonstrate that UPF1 exists in two essential viral RNPs during the late phase of HIV-1 replication: the first, in a nuclear export RNP that contains Rev, CRM1, DDX3 and the nucleoporin p62, and the second, which excludes these nuclear export markers but contains Gag in the cytoplasm. Interestingly, we observed that both UPF2 and the long isoform of UPF3a, UPF3aL, but not the shorter isoforms UPF3aS and UPF3b, are excluded from the UPF1-Rev-CRM1-DDX3 complex as they are negative regulators of vRNA nuclear export. *In* *silico* protein-protein docking analyses suggest that Rev binds UPF1 in a region that overlaps the UPF2 binding site, thus explaining the exclusion of this negative regulatory factor by HIV-1 that is necessary for vRNA trafficking. This work uncovers a novel and unique regulatory circuit involving several UPF proteins that ultimately regulate vRNA nuclear export and trafficking.

## 1. Introduction

The nucleocytoplasmic export of macromolecules (RNA and protein) and RNA-protein (RNP) complexes is one of the critical steps necessary to ensure normal cellular function. The study of retroviral RNA export has derived a great deal of information on this highly regulated process that represents a key step in the replication cycle that could serve as a viable therapeutic target [1,2]. For instance, the recruitment of viral and host factors to the unspliced viral genomic RNAs (vRNA is used herein) helps this molecule to overcome nuclear retention and promote nucleocytoplasmic export [3,4]. Bypassing host RNA surveillance machineries that target and clear unspliced and intron-containing, aberrantly spliced RNA substrates leads to the preservation of viral genomic RNAs and guarantees structural viral protein synthesis once they arrive in the cytoplasm [5,6,7].

While the nuclear RNA export factor 1 (NXF1) is required for the constitutive export of the 2-kb, completely-spliced viral RNA species [8], the 9-kb, unspliced, vRNA and the 4-kb, singly-spliced RNAs harbor the *cis*-acting Rev-responsive element (RRE) that are bound by the viral protein Rev to program nucleocytoplasmic RNA trafficking [9]. Many studies have highlighted the requirement for various host proteins in these transport processes. For example, eukaryotic translation initiation factor 5A (eIF5A) acts as an adaptor for Rev and the mammalian nuclear export factor Chromosomal Maintenance 1 (CRM1) [10,11]. RNA helicases such as DEAD box protein 1 and 3 (DDX1, 3) promote Rev-mediated export via interaction with CRM1 and Rev [12]. The Src-associated protein in mitosis, 68 kDa (Sam68), is not only required for Rev-dependent export, but is also involved in exporting RRE-containing RNAs in a Rev- and CRM1-independent manner [13,14]. Nuclear pore complex proteins, such as nucleoporin p62 (Nup62), also gate HIV-1 vRNA for nucleocytoplasmic export [15]. While these examples identify positive activators of nucleocytoplasmic export, insulin-like growth factor II mRNA binding protein 1 (IMP1) and HS1-associated protein X-1 (Hax-1) are host factors that negatively regulate Rev function during nuclear export [16,17]. Thus, the mechanisms and virus-host interactions surrounding these events represent viable targets to block HIV-1 gene expression and assembly [18].

UPF1 is an RNA-binding protein that possesses RNA helicase and ATPase activities, Zn-coordinating finger motifs as well as acidic and basic amino acid clusters [19,20]. It shuttles between the nucleus and cytoplasm via CRM1 and this characteristic imparts potential roles in both the nucleus and cytoplasm [21]. The roles of UPF1 are quite diverse in mammalian cells as reviewed in [22] and include RNA stability [23,24], DNA repair, cell cycle progression [25], DNA replication and telomere metabolism [26]. UPF1 exerts these functions in the context of several nuclear and cytoplasmic ribonucleoprotein (RNP) complexes [24,26,27,28,29,30,31]. Its best understood and characterized function lies in its role in nonsense-mediated mRNA decay (NMD), an RNA surveillance mechanism that targets mRNAs that harbour premature termination codons (PTC) for degradation [30,32]. NMD has been shown to regulate the stability of many physiological transcripts, thus implying an important role as a post-transcriptional regulator of gene expression, as reviewed in [19,33]. For example, miR-128 is a brain-specific miRNA that targets, among others, the UPF1 transcript leading to the repression of NMD and the concomitant upregulation of many proteins controlling neuronal development and function in differentiating neural cells [34]. The process of NMD has been shown to be tightly self-regulated to ensure that its regulatory and surveillance functions are not disturbed. As such, most transcripts encoding NMD factors are sensitive to NMD themselves [35,36]. The proteins involved in NMD include the UPF proteins (UPF1, UPF2 and UPF3) that form a complex that interacts with the exon-junction complex (EJC) via UPF3b [37,38]. This model is evolving, since new evidence suggests that UPF1 initially binds mRNAs before aberrant translation termination triggers NMD [39,40]. In addition, a UPF2-independent NMD pathway has also been described, obviating the requirement for UPF2 [41].

In addition to transcripts carrying a PTC, substrates for NMD include, among others, mRNAs harbouring long 3'-untranslated regions (UTR), introns in the 3'-untranslated region (3'-UTR) and frameshift sequences [33,39,42]. However, retroviruses are able to evade recognition by the NMD RNA quality control machineries, despite possessing very long 3'-UTRs [43]. Indeed, HIV-1 makes use of UPF1 during viral replication to promote vRNA stability in both nuclear and cytoplasmic compartments as well as vRNA translation in the cytoplasm, directly influencing steady-state levels of the main viral structural protein, Gag, and thus, virus production [5]. Recent findings suggest that UPF1 could exert this activity in part by a rapid association with the RNA during its *de* *novo* vRNA synthesis, immediately following transcription [44], observations consistent with the results presented herein (see below). In the cytoplasm, UPF1 assembles in a complex in which the vRNA, the viral structural protein Gag and host proteins Staufen1 and UPF3b are present but from which UPF2 is excluded [5,45]. All of these components are found in complex with UPF1 but the assembly into the HIV-1 RNP could also be mediated via an interaction with the vRNA [5,44]. It was also recently shown that UPF1 associates with the HIV-1 RNA in an RNA length-dependent manner [44,46].

UPF2 is a phosphoprotein that interacts with UPF1 and UPF3b to trigger NMD. In at least one study, the interaction between UPF2 and UPF1 was shown to be mediated by a conformational change of UPF1 from an RNA-binding to an RNA unwinding mode dampening UPF1’s ability to bind RNA [28]. The other component of the surveillance complex, UPF3, has two paralogs, UPF3a and UPF3b, and they both trigger NMD differently [47]. UPF3a has two isoforms, UPF3aL and an additional one called UPF3aS, which lacks exon 4 and binds UPF1 but not UPF2 [48]. Moreover, both UPF3aL and UPF3aS are found in different complexes: UPF3aS/PP2A/SMG5/SMG7 and phosphorylated UPF1 (P-UPF1) (called pre-dephosphorylation) and a complex lacking SMG5/7 but containing UPF2, UPF3aL and P-UPF1 (called post-phosphorylation) [31]. Moreover, UPF3b regulates UPF3aL protein levels, but this is dependent on their ability to associate to UPF2 [49]. This indicates that UPF3b and UPF3aL/S have differential roles in distinct RNPs. Moreover, P-UPF1 preferentially binds to both SMG5/7 and SMG6, and the knockdown of SMG6 results in an increase of UPF1 in complex with SMG5/7, thereby decreasing the abundance of UPF1 associated to UPF2-UPF3aL [31,50].

Since our earlier work strongly supported a nuclear role for UPF1 [5], in this report we characterize the importance of the nuclear interaction between UPF1 and the vRNA. We demonstrate that UPF1 shuttling promotes the nucleocytoplasmic export of vRNA. UPF1 expression also overcomes the nuclear retention of vRNA due to the absence of Rev expression. Importantly, using *in* *situ* imaging analyses and *in* *silico* modeling of protein-protein interactions, we revealed that the association between UPF1 and UPF2 plays a critical role in the regulation of vRNA nucleocytoplasmic export. These results concretely explain why HIV-1 excludes UPF2 from its nuclear export and cytoplasmic HIV-1 RNP but also identify an unsuspected regulatory circuit involving multiple host UPF proteins in determining the fate of the HIV-1 vRNA.

## 2. Methods

### 2.1. Cell Culture, Plasmids and Transfections

**Figure 1:**
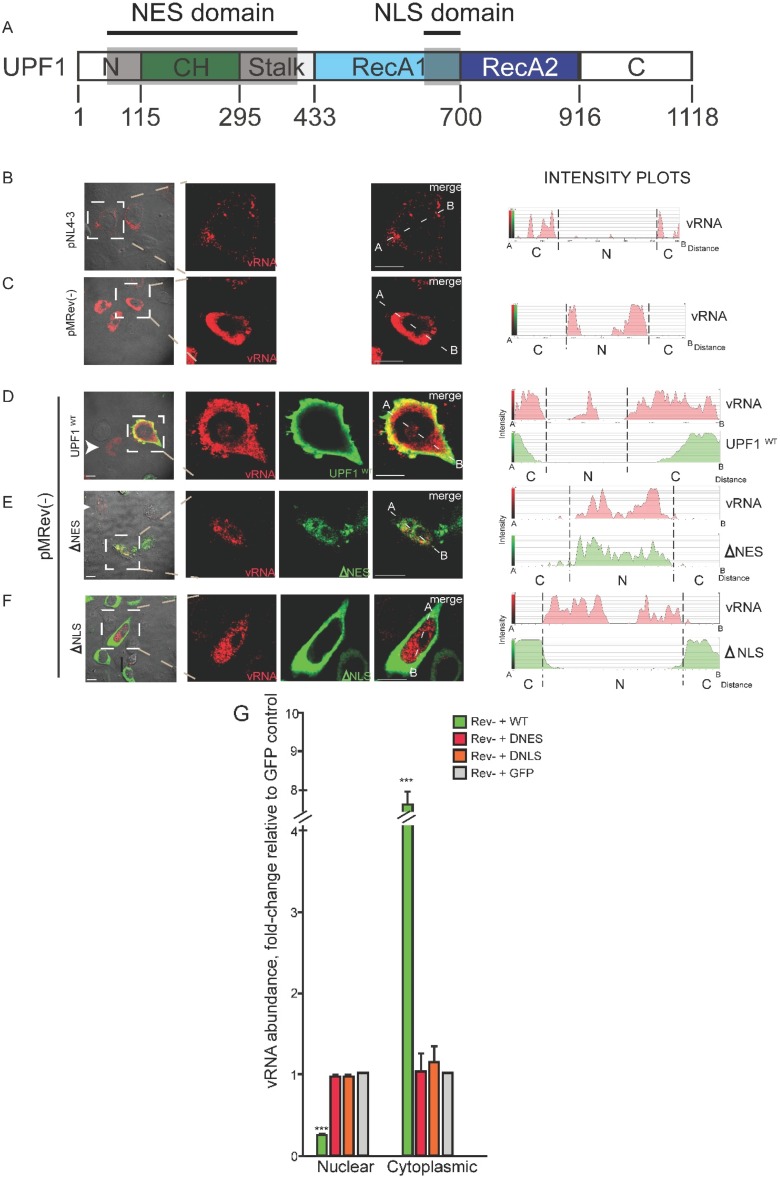
UPF1 nucleocytoplasmic shuttling promotes vRNA nucleocytoplasmic export (**A**); The UPF1 nuclear export and localization sequence (NES, NLS) domains are shaded in the depiction of UPF1. HeLa cells were co-transfected with wildtype proviral DNA (PNL4-3) alone (**B**); or a Rev-defective provirus pMRev(−) alone (**C**); or with UPF1^WT^ (**D**); UPF1^ΔNES^ (**E**); and UPF1^ΔNLS^ (**F**). vRNA shown in red and UPF1 proteins are shown in green (in all panels). White arrowheads identify HIV-1-transfected cells. Size bars indicate 10 µm. The corresponding Intensity plots for vRNA (red) and GFP-tagged UPF1 protein staining are shown from point A to B in the designate cells: dashed lines demarcate the nucleus (N) and cytoplasm (C) boundaries. Imaging results shown are representative of the phenotypes observed in >85% cells in each condition in 5 independent experiments. (**G**) Dunnett post-test analyses on vRNA localization results ±SEM values compared to the controls pNL4-3+GFP (pNL4-3) or GFP (Rev−); *** *p* < 0.001.

Cell culture of HeLa cells and transfections of proviral DNAs, pNL4-3 and pMRev(−) as well as UPF1 siRNA and rescue experiments were performed as described before [5]. Total cellular RNA was isolated by TriZol Reagent or TriZol LS (Life Technologies, Carlsbad, CA, USA) according to the manufacturer’s instructions. pMRev(−) construct was provided by the NIH AIDS Reference and Reagent Program (generously provided by Reza Sadaie). pCl-FLAG-UPF1 (UPF1^WT^) and pCI-FLAG were described earlier [5]. Bryan Cullen (Duke University, Durham, NC, USA) provided the HA-TapA17 plasmid [51] and Alan Cochrane (University of Toronto, Toronto, ON, Canada) provided the CTE-Gag plasmid. The following GFP-tagged UPF1 wildtype (UPF1^WT^) and UPF1 mutants were described earlier [21]. Because the NES has been defined as rather large, the ΔNES mutant lacks the nuclear export signal (NES) as well as both zinc fingers and ΔNLS lacks the RNA helicase domains II and III as well as the nuclear localization signal (NLS) (as shown in Figure 1A). Rev-R-YC was provided by Ruth Brack-Werner (GSF-National Research Center for Environment and Health, Neuherberg, Germany) [52]. FLAG-UPF3aS, FLAG-UPF3aL, FLAG-UPF3b, FLAG-UPF2^WT^, FLAG-UPF2^1173^ and FLAG-UPF2^1–1096^ were previously described [38,48,49,53,54]. FLAG-UPF3aS lacks exon4 from UPF3aL; FLAG-UPF2^1173^ has point mutations which abolish its interaction with UPF1; FLAG-UPF2^1–1096^ has a deletion at the C-terminus of the protein and does not bind UPF1 [47,48,53,54,55]. UPF proteins were overexpressed approximately 2–5-fold over western blotting signals obtained for endogenous UPF proteins. For the Leptomycin B (LMB) experiments, cells were transfected 20 h prior to collection and treated with 2 nM of LMB (Sigma-Aldrich, Oakville, ON, Canada) as described previously [56]. Monocytic OM10.1 cells were mock induced or induced with phorbol ester PMA and cell lysates were harvested for western analyses at the indicated time points. siRNA experiments for siNS and siUPF1 were performed as previously described [5].

### 2.2. Antisera and Reagents

Antisera to UPF1, UPF2 and UPF3b were generously supplied by Jens Lykke-Andersen (University of California, San Diego, CA, USA). Antisera to DDX3 was generously supplied by Luc DesGroseillers (University of Montreal, Montreal, PQ, Canada). Mouse anti-p24 and mouse anti-GAPDH antisera were purchased from Intracell and Techni-Science, respectively. Mouse anti-Digoxin was purchased from Sigma-Aldrich and sheep anti-Digoxin was purchased from Roche. Mouse and Rabbit anti-FLAG antibodies were purchased from Sigma-Aldrich and mouse anti-GFP was purchased from Roche. Rabbit anti-CRM1 was purchased from Santa Cruz. Rabbit and mouse anti-HA was purchased from Santa Cruz and Roche, respectively. The Nup62 antibody was purchased from Sigma-Aldrich and rat anti-RFP (identifying mCherry in IF analysis) was from Chromotek and the anti-SMG6 was from Abcam. For western analysis, horseradish peroxidase-conjugated anti-rabbit and anti-mouse antibodies were purchased from Rockland Immunochemicals (Pottstown, PA, USA). Signal intensities were scanned by densitometry using either ImageJ software (NIH, Bethesda, MD, USA) or using the Gel Doc System and quantitated by the Quantity One Software version 4.4.1 (BioRad, Montreal, QC, Canada) [57]. Standard curves were generated using cell extracts to ensure that signal intensities fell within the linear ranges of the assays using the antibodies described in the manuscript. For indirect IF and FISH, secondary fluorophore-conjugated antisera AlexaFluor donkey anti-mouse 488, 594 and 647, donkey anti-sheep 488 and 647 and donkey anti-rabbit 594 and 647 were purchased from Invitrogen [56].

### 2.3. FISH/IF Co-Analyses

Fluorescence *in* *situ* hybridization and immunofluorescence (FISH/IF) co-analyses were performed as described earlier [56,58,59,60]. The antisense RNA probe recognizes a 236bp long region in the pol gene (nt 1724–1960) and was prepared by *in* *vitro* transcription with digoxigenin-labeled UTP (Roche Applied Science, Montreal, QC, Canada). Microscopy was performed on a Zeiss LSM5 Pascal laser-scanning confocal microscope (LSCM) or on a WaveFX spinning disk confocal microscope system (Quorum Technologies, Inc., Guelph, ON, Canada). Microscopy techniques, filter sets and laser wavelengths were completely described earlier [56,58]. Quantitative imaging analyses were performed with the help of Imaris software v. 7.4 (Bitplane, Inc., Zurich, Switzerland). Intensity plots for antigen/vRNA signals was achieved using the measurement panel through the center focal plane, as described previously [58,61]. All imaging experiments were performed at least 3 and as many as 7 times with similar results. Nuclear *vs*. cytoplasmic phenotypes were defined only when >80% of the vRNA was localized in the nucleus or in the cytoplasm. Representative phenotypes are shown in the figures in the manuscript. The observed phenotypes were obtained in 78% to 85% cells (*n* > 50 cells per condition) in each experiment. Data were analysed by one-way analysis of variance followed by a Dunnett post-test using GraphPad Prism 5.0 software. This test compares the mean of each condition with that determined for the respective control. The histograms shown in the manuscript represent the fold-change in vRNA localization (±standard error of the mean, SEM) in either nuclear or cytoplasmic compartments with respect to control conditions. Quantitative evaluation of the localization was achieved by visual inspection of the central confocal plane of laser scanning confocal images; cells were excluded from the calculation when either the signal intensity for HIV-1 or UPF antigens was poorly detectable or when the distribution was not distinct such that it spanned the nucleus, nuclear periphery and cytoplasmic compartments. In most experiments, distinguishing the nucleus/cytoplasm limits was readily observable and in some experiments the use of the chromatin stain, DAPI or immunofluorescence of Nup62 was used to confirm the cytoplasm/nucleus boundary. Only cells in which HIV-1 and the co-expressed proteins were considered in the calculations, where applicable. A *p* value of <0.05 was considered statistically significant. The RNA-specific dye SYTO14 was used to localize bulk mRNAs in the study as described earlier [62] and the expression of the reporter gene mCherry (CMV-mCherry [63]; kindly provided by Paul Bieniasz, Aaron Diamond AIDS Center, New York, NY, USA) followed by biochemical cell fractionation analysis was used to assess the effects of UPF protein overexpression on mCherry and *mCherry* mRNA localization, a representative unrelated mRNA (to HIV-1 RNA). Overexpression of UPF constructs had little effect on bulk mRNA localization (see Supplemental Figure S1).

HeLa cells were transfected as described above and were described earlier [5]. For the FLAG immunoprecipitation, 1 mg of total lysate was incubated 2 h with agarose conjugated anti-FLAG beads and the bound complex was analyzed via SDS-PAGE analysis as described earlier [61]. For the Gag immunoprecipitation HeLa cells were transfected as described above. Cells were lysed in NP40 lysis buffer and Gag was immunoprecipitated as described previously using 1 mg of protein and affinity-purified mouse anti-p24 antisera (hybridroma 183-H12-5C) [64] from the NIH AIDS Reference and Reagent Program [5]. Signal intensities in immunoprecipitates were normalized to input protein levels found in total cell lysates and were quantitated by densitometry as described above. The lack of GAPDH immunoreactivity in the immunoprecipitates (output) demonstrated the specificity of this assay.

### 2.4. First STRAND cDNA Synthesis Followed by PCR

HeLa cells were transfected as described above and were described earlier [5]. For cell fractionation studies, the nuclear and cytoplasmic fractions were prepared as previously described prior to western analysis [15,65] and performed at least twice for each type of experiment. RNA was purified using TriZol and Trizol LS. 1 µg of total RNA was treated with DNAse I Amplification Grade (Life Technologies) and first strand cDNA synthesis was performed using Superscript II RT (Life Technologies). 2 µL of total cDNA was taken and PCR amplified for *gapdh* mRNA and vRNA using the primers described in Abrahamyan *et* *al*. [61]. *mCherry* mRNA was amplified using a specific primer pair (Forward primer, 5'-TGG AGG GCT CCG TGA ACG GCC and reverse primer 5'-TAG GCG CCG GGC AGC TGC ACG) to generate a RT-PCR product of about 483 bp. Signal intensities of PCR signals (cycle 25–30) were quantitated exactly as described above [61,62] using ethidium bromide stained DNA signals captured in-gel. All calculations were performed by taking into account the *gapdh* mRNA signal intensities.

### 2.5. Glycerol Gradient Fractionation of UPF1 Complexes

500 µg protein from lysates derived from cells expressing HIV-1 pNL4-3 and pCi-FLAG-UPF1 was immunoprecipitated using FLAG-M2 agarose beads (Sigma-Aldrich). Immunoprecipitates were eluted using FLAG-3X peptide as per the manufacturer’s protocol. The eluted FLAG-UPF1 complexes were then carefully layered on a 5%–50% glycerol gradient and centrifuged at 80,000× *g* for 2 h, 4 °C. 12–500 µL fractions were collected and the abundance of FLAG-UPF1, pr55^Gag^, CRM1 and UPF3b were characterized by Western blotting. The vRNA was extracted using TriZol according to the manufacturer (Invitrogen, Montreal, QC, Canada) and quantitated by RNA slot blotting analyses, exactly as described [66].

### 2.6. Protein-Protein Docking and Alanine Scanning

Protein-protein docking was performed using the structure of UPF1 (PDB: 2WJV) and DDX3 (PDB: 2I4I), previously described [53,67]. To obtain the structure of a Rev, we used the structure of the L12S/L60R mutant Rev dimer (PDB: 3LPH) and the structure of a wild type Rev monomer (PDB: 2X7L), previously described [68,69]. For this, we proceed with structural analysis between the dimer and monomer using the SALIGN module of MODELLER, version 9.13 [70]. We performed the protein-protein docking using the ZDOCK web server (Pierce, B.G. *et* *al*., 2014) with default parameters and without specifying amino acids for interaction. Then, the models with the best score were refined using the Rosseta server [71]. For alanine scanning, the Rossetta server was used and amino acids with ΔΔG ≥ 1 kcal/mol were defined as relevant for interaction as suggested [72]. Drawing of structures was performed with Pymol (The PyMOL Molecular Graphics System, Version 1.4.1 Schrödinger, LLC).

## 3. Results

### 3.1. UPF1 Shuttling Is Important for vRNA Nuclear Export

Our earlier work showed that the expression of either the wild-type UPF1 (UPF1^WT^) or the *trans*-dominant negative (TDN) NMD-null RNA helicase mutant, UPF1^R844C^, increased HIV-1 Gag and vRNA levels, indicating that UPF1’s function in vRNA metabolism is independent of its role in NMD [5]. While NMD does require an interaction between UPF1 and UPF2 in most circumstances [53], this association is not essential for the resulting effects on HIV-1 gene expression as UPF2 is excluded from a cytoplasmic Gag RNP [5] and UPF1 mutants that do not interact with UPF2 maintain Gag and vRNA upregulation. The effects of UPF1 are also exerted in the nuclear compartment, such that UPF1 depletion decreased steady-state levels of nuclear HIV-1 vRNA, but had little effect on the spliced RNA species, under Rev− conditions when vRNA is retained in the nucleus [5]. UPF1 overexpression in these conditions resulted in slight increases in Gag expression but more marked increases in vRNA (similar to that found in Rev+ conditions) (Supplemental Figure S2; [5]), suggesting that UPF1 promotes vRNA nuclear export in the absence of Rev but Rev expression appears to be important for Gag upregulation, as we have shown previously [62]. To determine the magnitude of the effect of UPF1 in the nucleocytoplasmic shuttling of the vRNA, we tested whether UPF1 could impact HIV-1 vRNA localization under Rev− conditions. We co-expressed GFP-tagged UPF1^WT^ and two shuttling mutants, one lacking a nuclear export signal (∆NES) and one lacking a nuclear localization signal (∆NLS; depicted in Figure 1A) with proviral DNA pMRev− and examined HIV-1 vRNA localization. In this manuscript, vRNA is detected by sensitive fluorescence *in* *situ* hybridization/immunofluorescence (FISH/IF) co-analyses coupled with laser scanning confocal microscopy (LSCM) [59,60]. As expected, the expression of the Rev-deficient provirus pMRev(−) prevented vRNA export as most of the signal was observed in the cell nucleus (compare Figure 1B,C; and accompanying intensity plots). Surprisingly, the ectopic expression of UPF1^WT^ resulted in the translocation of the vRNA to the cytoplasm as evidenced by the prominent cytoplasmic signal of the vRNA (Figure 1D). In sharp contrast, the expression of the shuttling mutants of UPF1, UPF1^ΔNES^ and UPF1^ΔNLS^, did not promote vRNA nuclear export (Figure 1E,F). The relocalization of the HIV-1 vRNA under Rev− and UPF1 overexpression conditions correlates with the increase in Gag expression observed (Supplemental Figure S2) and was corroborated in subcellular fractionation assays (Supplemental Figure S1). This ectopic expression of UPF1 may impact on the genomic RNA at several levels (see Discussion).

The effect of UPF1 on vRNA localization was specific as we did not observe any major changes in total mRNA distribution upon expression of UPF1 as determined by staining with the dye, SYTO14 (Supplemental Figure S3A). Moreover, UPF1 overexpression did not affect the localization of mRNA and protein levels of the unrelated mCherry (Supplemental Figure S3B (compare lanes 5 and 8 with 6 and 9, respectively) Statistical Dunnett post-test analyses on vRNA localization patterns compared to control levels (Rev- and GFP control vector) showed that the overexpression of UPF1^WT^ promoted a significant translocation of vRNA to the cytoplasm in Rev− conditions (Figure 1G). Although the expression of UPF1^ΔNES^ had little effect on vRNA localization (Figure 1E and corroborated in subcellular fractionation experiments, Supplemental Figure S1), this mutant UPF1 maintained an interaction with the vRNA, whereas UPF1^ΔNLS^ did not, indicating that the vRNA:UPF1 interaction likely initially occurs in the nuclear compartment [73]. These results demonstrate that the shuttling activity of UPF1 is important for directing HIV-1 vRNA to the cytoplasmic compartment. The characteristics and binding partners of UPF1^WT^, UPF1^ΔNES^ and UPF1 ^ΔNLS^ as well as a summary of their effects on Rev− HIV-1 vRNA export are summarized in Table 1.

**Figure 2:**
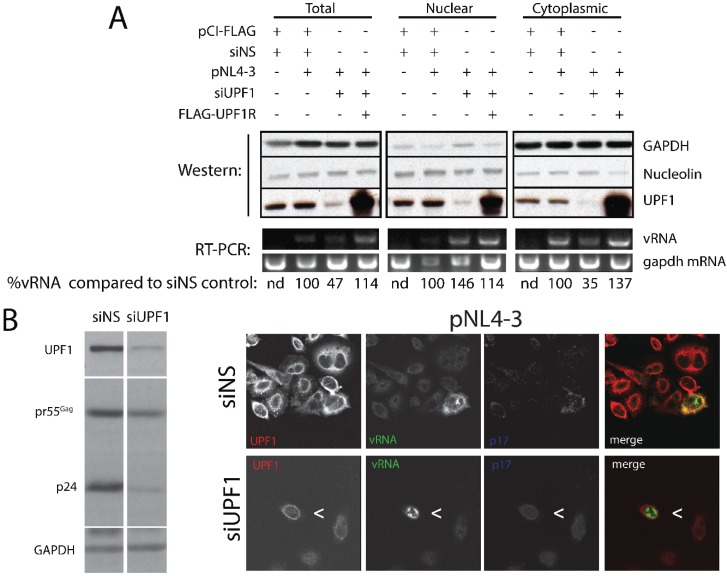
UPF1 depletion increases the abundance of vRNA in the nucleus while UPF1 Rescue enhances cyto-plasmic vRNA levels. (**A**) HeLa cells were mock transfected with pCI-FLAG and non-silencing siRNA (siNS) or co-transfected with HIV-1 proviral pNL4-3 DNA and pCI-FLAG and siNS. UPF1 was depleted by siUPF1 and UPF1 expression was rescued with FLAG-UPF1Rescue (UPF1R). At 30h post-transfection, cells were harvested and cytoplasmic-nuclear fractionation analysis was carried out as described in Materials and Methods. Western blot analysis was performed GAPDH (enriched in the cytoplasmic), Nucleolin (enriched in the nucleus), and UPF1 (identifying endogenous and overexpressed proteins). RNA was isolated from cell extracts and steady-state levels of vRNA and gapdh mRNA was measured by RT-PCR. vRNA levels were quantified by densitometry with ImageJ using gapdh mRNA signal intensity for normalization; pNL4-3 + siNS condition was set at a 100%. nd, not detectable. (**B**) Cells were mock-depleted with nonsilencing siRNA (siNS) or depleted using siUPF1 as described in Materials and Methods. At 40–48 h post-transfection, cells were harvested for western blotting for UPF1 (endogenous), Gag (identifying pr55Gag and p24) and GAPDH (loading control) (on left) and FISH/IF co-analyses for UPF1 (red), vRNA (green) and Gag (blue) (with merged renditions on right). UPF1 depletion led to decreased Gag expression as shown earlier (left panel; Ajamian *et* *al*., 2008) but also led to a blockade to vRNA nuclear export (white arrowhead < in lower row of right panel) in >80% cells examined (*n* = 48). See results for additional discussion of these findings.

In earlier work, we showed that the abundance of vRNA correlated directly with the UPF1 expression levels in both Rev+ and Rev− conditions [5] supporting a role in vRNA stability. In addition, rescuing UPF1 following siRNA-mediated depletion led to a complete recovery of HIV-1 Gag synthesis but vRNA was not completely rescued, demonstrating that UPF1 also acted on vRNA translation [5]. Here, we determined how UPF1 depletion influenced the localization of HIV-1 vRNA. To do this, we depleted UPF1 and then rescued expression and performed subcellular fractionation and identifed nuclear and cytoplasmic marker proteins. Upon UPF1 depletion, vRNA markedly partitioned to the nuclear compartment with a concomitant 3-fold decrease in abundance in cytoplasmic levels (Figure 2A). We then performed a partial knockdown of UPF1, since the depletion of UPF1 usually leads to below-threshold detection of vRNA [5]. In cells with detectable vRNA signals, FISH/IF co-analyses showed that HIV-1 vRNA accumulated or was retained in the nucleus upon UPF1 depletion in >80% cells (*n* = 48; Figure 2B). This was a rarely captured observation indeed, because vRNA levels are highly sensitive to steady-state UPF1 expression levels [5]. These data indicate that a deficiency in UPF1 leads to the nuclear retention of vRNA.

**Table 1:** UPF1 plasmids cited in this report.

UPF1 Plasmids	Deleted Region	Protein Localization	Characteristics	Effect on HIV-1 RNA Export (Rev− Conditions)	Interactions	
					UPF2	Staufen1
UPF1^WT^	NA*	Shuttling [21]	Binds UPF3a [48]; UPF3b [48]; Staufen1 [24,74]; UPF2 [48,74]; Nup100/116 (hNup98) [75]; nuclear HIV-1 vRNA [44]; and found in HIV-1 [61] and SupT1 and THP1 HIV-1 cores [76]	Positively affects export under Rev− conditions [5]; (this study)	Yes	Yes
UPF1^ΔNES^	55–416	Nuclear [21]	Lacks nuclear export signal domain, zinc fingers (CH domain (115–295) [21,77]; required for UPF2 and Staufen1 binding [74]	No effect under Rev− conditions (this study)	No	No
UPF1^ΔNLS^	596–697	Cytoplasmic [21]	Lacks nuclear localization signal domain, helicase domains II and III [21,77]	No effect on Rev− export (this study)	Yes	Yes

* NA: not applicable.

### 3.2. The CRM1-Mediated Export Pathway Is Favoured by UPF1 for Efficient vRNA Export

**Figure 3:**
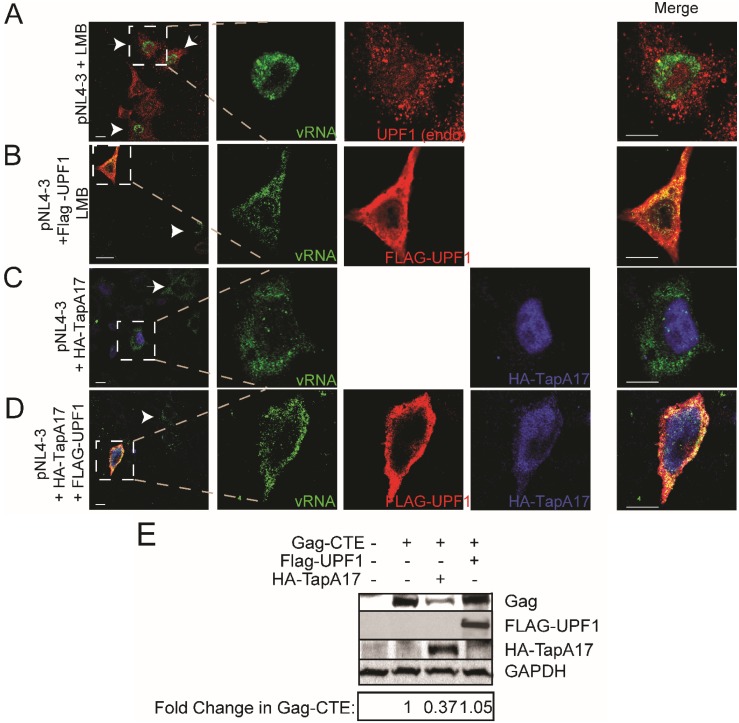
UPF1 exports vRNA via CRM1 and not the NXF1 export pathway. HeLa cells were transfected with pNL4-3 alone and treated with LMB (**A**); co-transfected with Flag-UPF1 and treated with LMB (**B**); co-transfected with HA-TapA17 (**C**); or co-transfected with HA-TapA17 and FLAG-UPF1 (**D**). FISH/IF co-analyses to identify the localization of vRNA (green in all rows), either endogenous UPF1 (red, A) or FLAG-UPF1 expressed in trans (red, B and D) or HA-TapA17 (blue in C and D). Merged renditions are shown on right-most panels. White arrowheads identify cells exhibiting LMB-induced block to nuclear vRNA export in (in A and B) and cells expressing HIV-1 (in C and D). Imaging results are representative of the phenotypes observed in 78%–85% cells in each condition in 3 experiments. Size bars, 10 µm; (**E**) Western blot analysis for Gag, FLAG-UPF1, HA-TapA17 and GAPDH (loading control). ImageJ was used to quantify the relative expression levels of Gag (normalized to GAPDH levels). Results shown represent averages from 2 independent experiments with <10% deviation between experiments. nd: not detectable.

The two essential nucleocytoplasmic export pathways are those mediated by CRM1 and NXF1. HIV-1 uses both export pathways for the export of its different RNA transcripts such that multiply-spliced RNAs are constitutively exported via NXF1 while Rev-dependent transcripts including the vRNA, are exported in a regulated manner to the cytoplasm via CRM1 [9]. To differentiate between the use of these two export pathways mediated by UPF1, FISH/IF co-analyses were employed to visualize the localization of the vRNA under a pharmacologic block of CRM1 mediated nuclear export using Leptomycin B (LMB) or a block of NXF1 mediated nuclear export imposed by the expression of a negative transdominant NXF1 (HA-TapA17) [51]. In contrast to the typical cytoplasmic localization of vRNA from the wild type provirus (Figure 1B), LMB treatment blocked Rev-mediated nucleocytoplasmic export resulting in the accumulation of vRNA in the nucleus (Figure 3A, shown in green). LMB also blocked CRM1-mediated shuttling of endogenous and, in part, exogenously-expressed FLAG-tagged UPF1 that lead to a partial accumulation in the nucleus in several experiments ((Figure 3A,B; UPF1 is shown in red in this figure) as previously shown [21]). In contrast, a normal cytoplasmic and punctate distribution of the vRNA was observed when NXF1-mediated export was blocked using HA-TapA17 in Rev+ conditions and with FLAG-UPF1^WT^ overexpression (Figure 3C,D). UPF1 also exhibited a normal cytoplasmic localization at steady-state upon expression of HA-TapA17 (Figure 3D). Since the vRNA as well as a fraction of UPF1 was trapped in the nucleus when CRM1-mediated export was blocked, but not when NXF1-mediated export was blocked, we conclude that UPF1 promotes vRNA nucleocytoplasmic export primarily via the CRM1 export pathway.

To validate specificity, we determined if this newly characterized export function of UPF1, which is CRM1-dependent in the context of *bona* *fide* HIV-1 expression, is also required for CTE-directed export of *gag* mRNA. CTE is the constitutive transport element which serves as a nuclear export signal for unspliced viral RNA of type D retroviruses by binding to the cellular mRNA export factor NXF1. Therefore, we co-expressed Gag-CTE with either FLAG-UPF1^WT^ or HA-TapA17 and assessed Gag expression levels by western blotting (Figure 3E). The overexpression of HA-TapA17 blocked NXF1-mediated export by 80%–90% and resulted in a decrease in Gag-CTE expression, while importantly, there was no change to Gag-CTE levels when FLAG-UPF1^WT^ was expressed (Figure 3E). These results show that while UPF1 can promote the export of HIV-1 vRNA destined for CRM1 export, it cannot enhance CTE/NXF1-mediated export, at least in the context of Gag.

**Figure 4:**
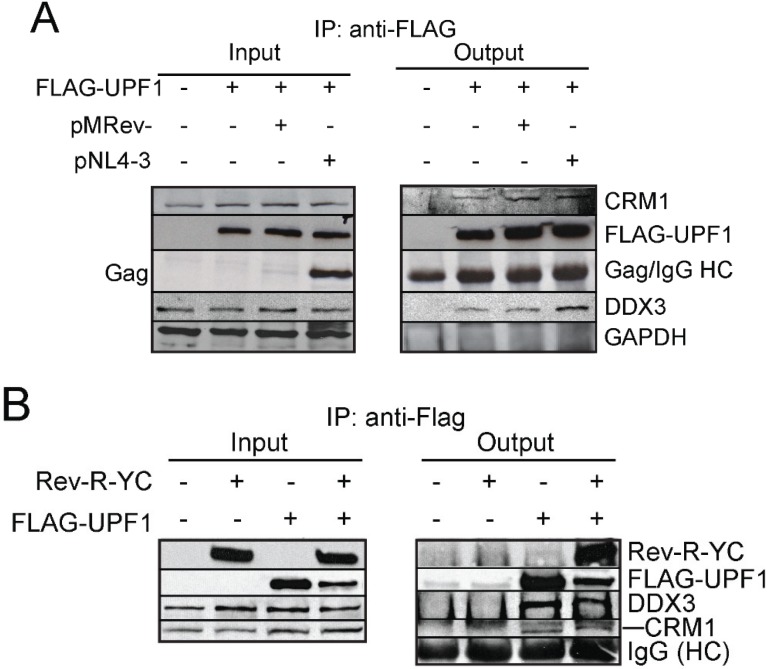

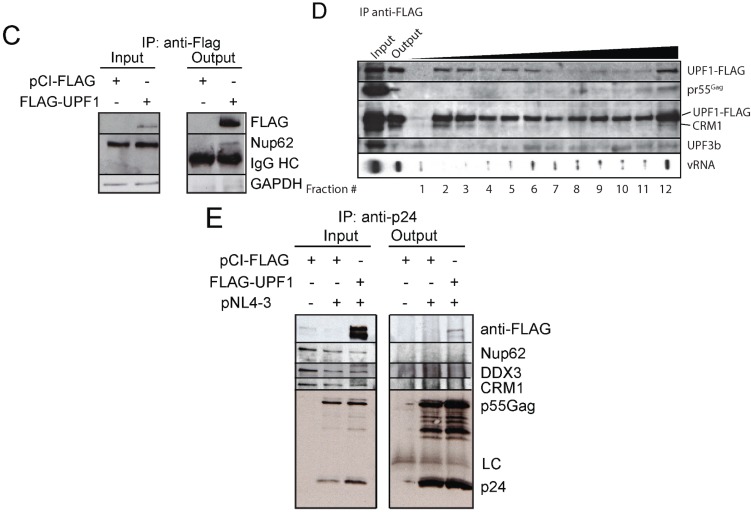
UPF1 is found in complex with Rev-CRM1-DDX3-Nup62. (**A**) HeLa cells were mock transfected with pCI-FLAG or transfected with FLAG-UPF1 alone or with either pMRev− or Rev+ pNL4-3 DNAs. FLAG-UPF1 was immunoprecipitated as described in Materials and Methods. Input lysates and bound complexes were analyzed by western blotting for CRM1, FLAG-UPF1, Gag, DDX3 and GAPDH (loading and output control). HC: Heavy Chain IgG. Results shown are representative of three independent experiments; (**B**) HeLa cells were mock transfected with pCI-FLAG or FLAG-UPF1 or co-transfected with FLAG-UPF1 and Rev-R-YC. FLAG-UPF1 was immunoprecipitated and western blotting of input lysates and bound complexes for Rev, FLAG-UPF1, DDX3 and CRM1. HC: Heavy Chain IgG; (**C**) HeLa cells were mock transfected with pCI-FLAG or transfected with FLAG-UPF1. Cells were harvested and FLAG-UPF1 was immunoprecipitated as described in Materials and Methods. Western blotting analysis for FLAG-UPF1 and Nup62. IgG HC: IgG heavy Chain; (**D**) Glycerol gradient analyses of UPF1 complexes in HIV-1-expressing cells. HIV-1/FLAG-UPF1-expressing cells were immunoprecipitated with anti-FLAG serum. FLAG-UPF1 complexes were eluted and fractionated on a glycerol gradient. Fractions were collected and FLAG-UPF1, pr55^Gag^, CRM1 and UPF3b were probed in each fraction by Western blotting. vRNA abundance is shown on the bottom panel, as determined by slot blot analyses; (**E**) HeLa cells were mock transfected with pCI-FLAG or co-transfected with HIV-1 and FLAG-UPF1. At 30 h post--transfection, Gag was immunoprecipitated using monoclonal anti-p24 antisera. Western for UPF1 (FLAG), Gag, CRM1, Nup62 and DDX3 were performed. Light Chain IgG.

HIV-1 encodes the regulatory viral protein Rev to export singly-spliced RNAs and the unspliced, vRNA to the cytoplasm [9]. Rev binds to the vRNA via an interaction between its NLS domain and the RRE element of the vRNA directing it through the nuclear pore via an interaction with CRM1 and nucleoporins [10,78,79]. Because our results highlight the importance of the shuttling function of UPF1 in vRNA export in both Rev-dependent and -independent conditions, we attempted to identify if UPF1 was present in the RNP complex required for vRNA nucleocytoplasmic export. To do this, we overexpressed FLAG-UPF1^WT^ with or without a Rev expressor (Rev-R-YC) and immunoprecipitated FLAG-UPF1^WT^. Rev, DDX3 and CRM1 all co-immunoprecipitated with UPF1^WT^ in the presence and absence of Rev (Figure 4B). In earlier published work, we showed that the depletion of Nup62 resulted in a block to vRNA nuclear export and Nup62 was also found to be part of the HIV-1 Rev RNP complex [10,15]. We therefore sought to determine if UPF1 could also co-precipitate with Nup62. Indeed, Nup62 was also found to be part of the UPF1 RNP strengthening the role of UPF1 in vRNA export (Figure 4C). In order to reinforce the notion that UPF1 is involved in vRNA export and is present in two different complexes, we also performed glycerol gradient analysis on immunoprecipitated UPF1 complexes in HIV-1 positive conditions and found that in lighter fractions UPF1 is associated with CRM1 and the HIV-1 vRNA and in the more dense fractions it is associated with CRM1, UPF3b, Gag and vRNA, similar to the Staufen1-HIV-1 RNP complex [5,45,80] (Figure 4D). In addition, DDX3, CRM1 and Nup62 were all absent from the HIV-1 Gag RNP complex as determined by co-IP analysis using anti-p24 [26] (Figure 4E) suggesting that UPF1 is found in at least two different HIV-1 RNPs: the first being composed of the vRNA and nucleocytoplasmic export factors such as CRM1, Rev, Nup62 and DDX3, most likely a nuclear RNP complex, and a second UPF1 cytoplasmic complex that includes Gag and a variety of other cytoplasmic proteins likely to be important for viral assembly, as we have proposed [5].

### 3.3. A Model for the Assembly of the UPF1/DDX3/Rev Complex

**Figure 5:**
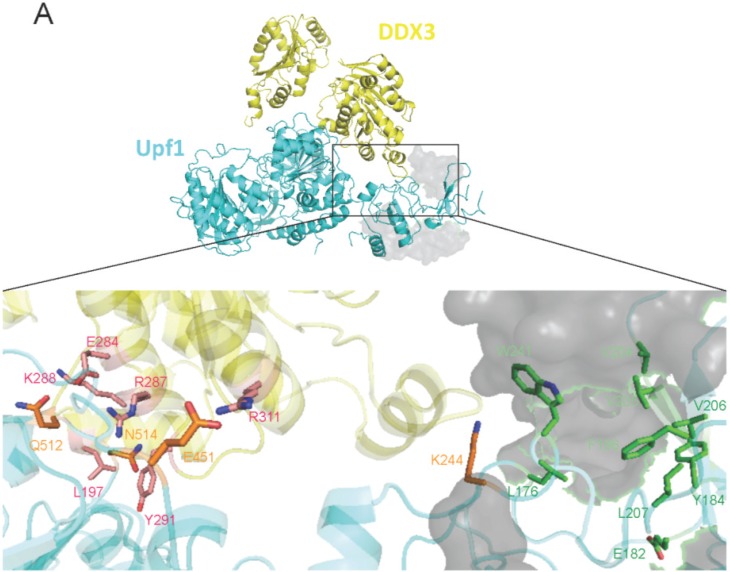

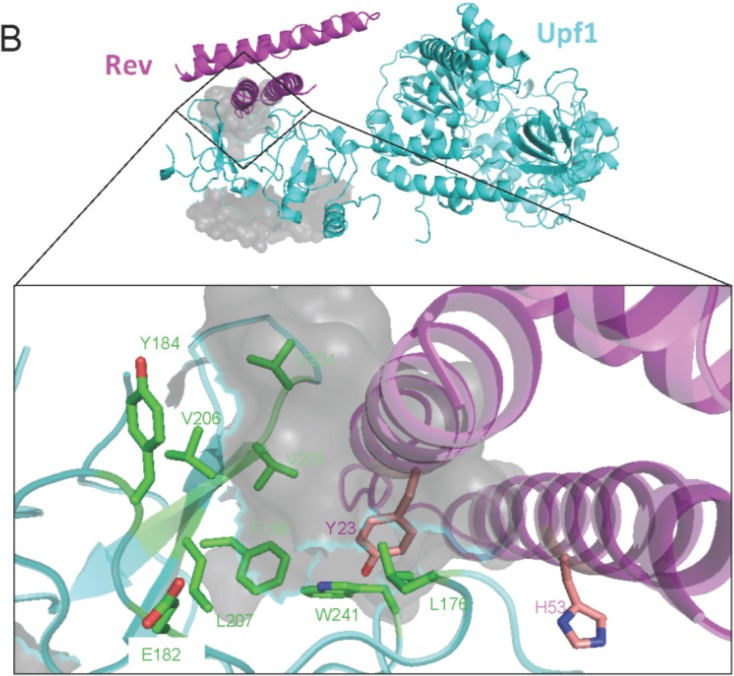
Interaction models and a mechanism for UPF2 exclusion. (**A**) Structural model for the interaction between UPF1 (cyan) and DDX3 (yellow) and structural alignment with the UPF1-UPF2 complex. UPF2 is represented as a gray surface (upper). An amplified image of the model for the UPF1-DDX3 complex is presented (bottom). Relevant residues obtained for UPF1 (orange) and DDX3 (pink) through alanine scanning or described in previous work as relevant for interaction with UPF2 (green) are shown as sticks. (**B**) Structural model for the interaction between UPF1 (cyan) and Rev (magenta) and structural alignment with the UPF1-UPF2 complex. UPF2 is represented as a gray surface (upper). An amplified image of the model for the UPF1-Rev complex is presented (bottom). Relevant residues obtained for UPF1 (orange) and Rev (pink) through alanine scanning or described in previous work as relevant for interaction with UPF2 (green) are shown as sticks. Drawing was performed with the Pymol software as described in materials and methods.

The results presented above show the formation of a multimeric complex containing UPF1, DDX3, CRM1 and Rev is necessary for nuclear export of the HIV-1 vRNA. UPF1 might bind to CRM1 through its NES as UPF1^ΔNES^ is retained in the cell nucleus. On the other hand, DDX3 and Rev also bind directly to CRM1 through the helicase core domain and a NES, respectively [12,81]. However, details on the UPF1-DDX3 or the UPF1-Rev interaction are unknown. Thus, in order to characterize the interactions inside this particular RNP complex assembled during HIV-1 replication, we generate computational models through protein-protein docking (Figure 5). For this, we took advantage of previously described structures of UPF1 in the context of the UPF1-UPF2 complex [53] and of DDX3 in complex with AMP-PNP [67]. In order to obtain the structure of a wild type Rev dimer, we used the structure of the wild type monomer [68] and the structure of the dimer containing the L12S and L60R mutations [69]. It should be mentioned that the structure of the Rev dimer used for modeling lacks the NES and is not bound to the RRE. However, the NES was predicted to be projected away from the arginine-rich motif (ARM) and thus, may not interfere with protein folding [69]. Structural models for UPF1-DDX3 (Figure 5A) and UPF1-Rev (Figure 5B) were generated using the ZDOCK web server [82] and the models with the best score were refined with the ROSSETA server [71] as described in Materials and Methods.

In the UPF1-DDX3 complex, we observed a wide surface of contact between the RecA-like domain 1 of DDX3 and the RecA-like domain 1A and the CH-domain of UPF1 (Figure 5A, upper panel). A structural alignment between the modeled structure of the UPF1-DDX3 complex and the structure of the UPF1-UPF2 complex [53] indicates that there is no apparent interference between DDX3 and UPF2 for binding UPF1 (Figure 5A, upper panel). Then, to understand the interaction between UPF1 and DDX3 at the molecular level, we perform an *in* *silico* alanine scanning using the Rosetta server as described in materials and methods [72]. We identified residues in the UPF1 Rec-like domain 1A domain (E451, N514 and Q512) and CH-domain (K244) as relevant for the interaction with DDX3 (Figure 4A, bottom panel). We also identified residues L197, E284, R287, K288, Y291 and R311 in the Rec-like domain 1 of DDX3 as relevant for the interaction with UPF1 (Figure 5A, bottom panel). Residues in UPF1 previously described as critical for its interaction with UPF2 [53,83] are shown in green (Figure 5A, bottom panel). Of note, our alanine scanning also identified UPF1 residue W241, a residue involved in UPF2 binding [53,83] as relevant for interaction with DDX3 suggesting a partial overlap between DDX3 and UPF2 that could interfere with binding of the latter (Figure 5A, bottom panel).

In the Rev-UPF1 model, we observed that one of the monomers of Rev interacts with the CH-domain of UPF1, a region described as critical for interaction with UPF2 [53,83]. Indeed, our structural alignment revealed that Rev could bind UPF1 at the same region that binds the β-strand of UPF2 (Figure 5B, upper panel). Such a secondary structure in UPF2 was described as the most relevant for its interaction with UPF1 as mutations in the β-strand results in the disassembly of the UPF1-UPF2 complex [53,83]. Interestingly, two residues in UPF1 important for its interaction with UPF2 (W241 and L176) were identified by alanine scanning as relevant for Rev binding (Figure 5B, bottom panel). Moreover, our model also shows that additional residues in UPF1 that are relevant for its interaction with UPF2 such as F196, V204 and V05 could be completely masked by Rev (Figure 5B, bottom panel). Despite the limitations of our modeling analyses, it appears that in the context of HIV-1 infection, Rev could interfere with the formation of an UPF1-UPF2 complex, without interfering with the UPF1-DDX3 interaction. This nicely correlates with our previous work indicating that UPF2 is not required for the UPF1-mediated stimulation of HIV-1 expression and provides molecular insights into the how UPF2 is excluded from the UPF1 complex assembled during HIV-1 replication [5,45] (and see below). Nevertheless, biochemical analyses using mutants are required to validate the residues that are important for the UPF1-DDX3 and UPF1-Rev interaction.

### 3.4. HIV-1 Induces the Exclusion of UPF2 from UPF1 Complexes

**Figure 6:**
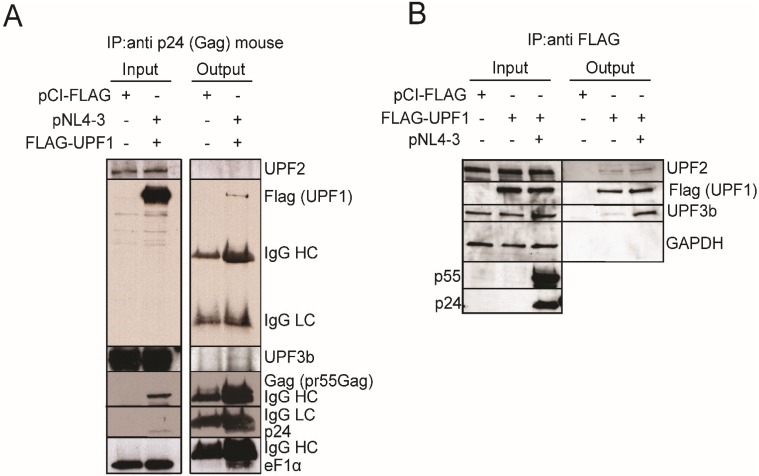
UPF2 is absent from the HIV-1 Gag RNP and UPF3b co-immunoprecipitates with UPF1 under HIV-1 conditions. (**A**) HeLa cells were mock transfected with pCI-FLAG or co-transfected with proviral pNL4-3 DNA and FLAG-UPF1^WT^. At 30 h post-transfection, cells were harvested and Gag was immunoprecipitated using a monoclonal anti-p24 antiserum as described in Materials and Methods. Western blot analysis for FLAG-UPF1, UPF3b, Gag (pr55^Gag^), UPF2, and eF1α (positive control) was performed. HC: IgG Heavy Chain. LC: IgG Light Chain. Results are representative of two experiments. (**B**) HeLa cells were mock transfected with pCI-FLAG or FLAG-UPF1 or co-transfected with HIV-1 pNL4-3 DNA. Cells were harvested and FLAG-UPF1 was immunoprecipitated. Input lysates and bound complexes were analyzed by western blotting for UPF2, FLAG-UPF1, UPF3b, GAPDH (loading and output control) and Gag (pr55^Gag^). Results shown are representative of three independent experiments. HC: IgG Heavy Chain, LC: IgG Light Chain.

The UPF1-3 proteins are components of different RNPs. For example, UPF1 exists in either UPF2-containing or UPF2-free NMD complexes. P-UPF1 is in complex with either UPF3aS/PP2A/SMG5/SMG7 or UPF3aL/UPF2 [31]. We therefore wanted to determine if there was a change in the association between UPF1, UPF2 and UPF3b in HIV-1 expressing cells, especially since our earlier work demonstrated that UPF2 was excluded from the HIV-1 RNP and we identified UPF3b in this RNP even though its overexpression did not increase HIV-1 vRNA levels [5]. Thus, HeLa cells were transfected with or without proviral DNA pNL4-3 and with UPF1^WT^. Gag was immunoprecipitated and the presence of UPF1, UPF2 and UPF3b was assessed in the immunoprecipitate (Figure 6A). The results show that UPF1 co-immunoprecipitated with Gag as previously reported, while UPF2 was absent from the Gag RNP (Figure 6A). UPF3b was also (weakly) present in the Gag RNP complex (Figure 6A). These results are consistent with our previous observations that showed that both UPF1 and UPF3b were present in the Staufen1-HIV-1 RNP complex, while UPF2 was absent [5,45]. We then determined if HIV-1 modulated the interactions of UPF proteins. HeLa cells were transfected with FLAG DNA (empty vector pCI-FLAG) or wild type UPF1 (FLAG-UPF1^WT^) with or without proviral DNA pNL4-3. FLAG complexes were then immunoprecipitated and the eluted complexes were assessed for FLAG (UPF1), UPF2, UPF3b, Gag and GAPDH by Western blotting analysis (Figure 6B). We observed that UPF3b and UPF2 co-immunoprecipitated with UPF1^WT^ but UPF3b co-immunoprecipitated with UPF1 to a several-fold greater extent in HIV-1 conditions in which little change in the association between UPF1 and UPF2 was observed. These observations indicate that the interaction between UPF3b and UPF1 is promoted and favoured by HIV-1, in contrast to that found to occur between UPF1 and UPF2, which is not modulated by HIV-1 and could explain the exclusion of UPF2 from the HIV-1 RNP [5].

### 3.5. UPF2, in Complex with UPF1, Blocks vRNA Nucleocytoplasmic Export

**Figure 7:**
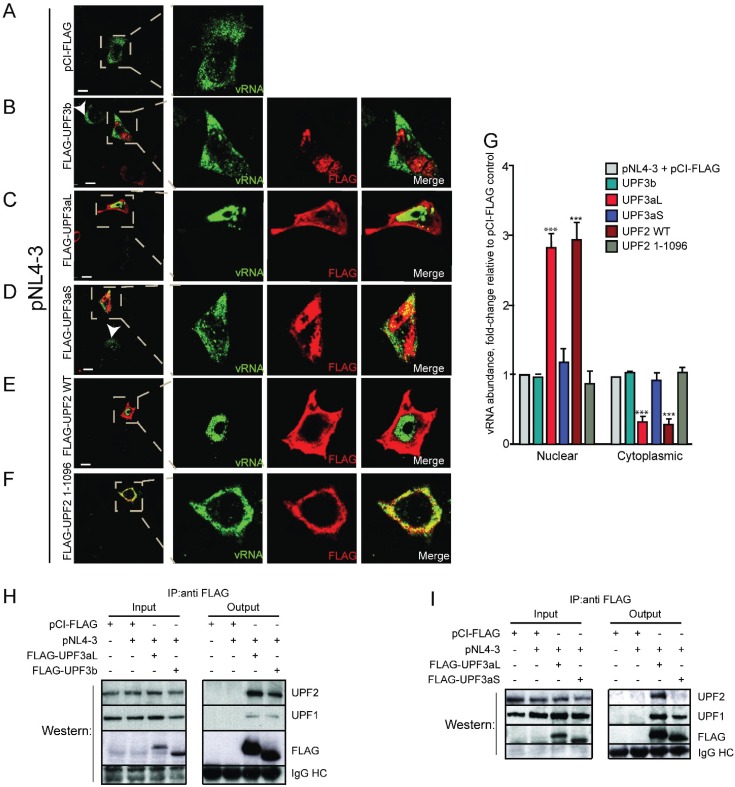

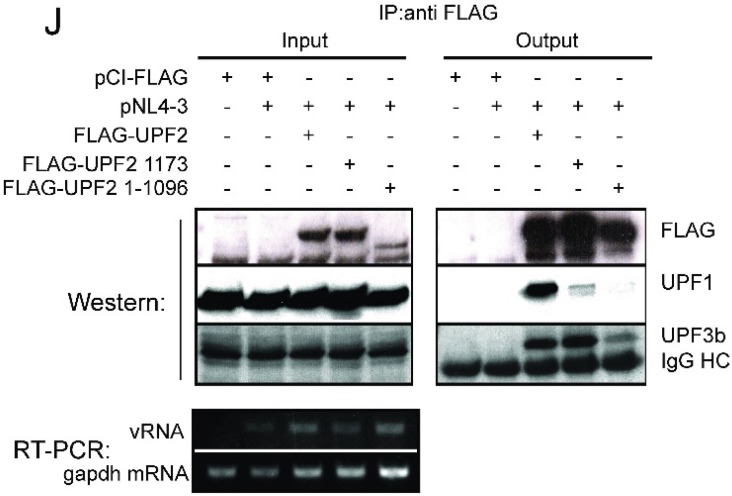
UPF3aL and UPF2 block vRNA export by associating to UPF1 as determined by FISH/IF and co-immunoprecipitation analyses. HeLa cells were co-transfected with pNL4-3 with pCI-FLAG (**A**); and FLAG-tagged UPF proteins: UPF3b (**B**); UPF3aL (**C**); UPF3aS (**D**); UPF2 (**E**); or UPF2 1-1096 (**F**); and processed for FISH/IF co-analyses to determine the localization of vRNA (green) and UPF proteins (**B**–**F**) (red); White arrowheads identify cells transfected with pNL4-3 alone. (**G**) Dunnett post-test analyses on vRNA localization results ±SEM values compared to the controls pNL4-3+pCI-FLAG: *** *p* < 0.001. Co-immunoprecipitation analyses confirm that UPF2 association blocks vRNA export. HeLa cells were mock transfected with pCI-FLAG or co-transfected with HIV-1 pNL4-3 proviral DNA and pCI-FLAG or FLAG-UPF3aL or FLAG-UPF3b (H). HeLa cells were transfected as in (**H**) but FLAG-UPF3aS was included instead of FLAG-UPF3b (**I**). HeLa cells were transfected as in (**H**) except that FLAG-tagged UPF2 proteins were co-transfected: UPF2WT or UPF21173 or UPF21-1096 (**J**). At 48 h post-transfection, cells were harvested and FLAG-tagged UPF proteins were individually immunoprecipitated as described in Materials and Methods. Input and bound (Output) complexes were analyzed by western blotting for UPF proteins as indicated. The abundance of vRNA and *gapdh* RNA was estimated by RT-PCR (in Input only). Results shown are representative of 2 independent experiments.

The major binding partners of UPF2 are UPF1, UPF3aL and UPF3b. Our previous results demonstrate that UPF3b overexpression did not elicit any changes to vRNA or Gag levels, and UPF2-binding mutants of UPF1 still upregulated Gag levels [5]. In addition, HIV-1 vRNA localized in a punctate pattern in the cytoplasm of cells (Figure 7A) consistent with our earlier work [5] and UPF3b expression had no marked effect on this localization pattern (Figure 7B). Since it was shown that UPF3aL and UPF3b undergo a unidirectional regulation that is dependent on their binding to UPF2 [49] and we had previously shown that UPF3b had no effect on vRNA metabolism [5], we wondered whether overexpressed UPF3aL could have a role similar to its paralog, UPF3b. To our surprise, UPF3aL expression blocked vRNA nucleocytoplasmic export resulting in a phenotype similar to that found when Rev is absent (Rev−, Figure 7C; compare with Figure 1C), in contrast, to the localization phenotype obtained with UPF3b (Figure 7B). Since we have shown that UPF1 is involved in vRNA export, we determined how these UPF proteins bound UPF1. HeLa cells were transfected with FLAG DNA (empty vector pCI-FLAG) with or without proviral pNL4.3 and with UPF3aL (FLAG-UPF3aL) or UPF3b (FLAG-UPF3b) with proviral pNL4-3. FLAG complexes were then immunoprecipitated with FLAG-conjugated agarose beads and the eluted complexes were assessed for FLAG, UPF1 and UPF2 by western blotting analysis. UPF2 and UPF1 co-immunoprecipitated with both UPF3b and UPF3aL (Figure 7H), without any change in vRNA levels [73].

UPF3b and UPF3aL undergo a unidirectional regulation which is dependent on the availability of UPF2. Since UPF2 is excluded from the HIV-1 RNP, we hypothesized that UPF2 binding to UPF3aL with UPF1 mediates the blockade to vRNA nuclear export. We therefore overexpressed the UPF3aL mutant, FLAG-UPF3aS, that does not interact with UPF2 but is a component of a complex containing UPF1 (but not UPF2), and examined vRNA localization by FISH/IF co-analyses (Figure 7D). In contrast with what was observed with UPF3aL, overexpression of FLAG-UPF3aS did not lead to the nuclear localization of the vRNA (Figure 7D). We then overexpressed FLAG-UPF3aL and FLAG-UPF3aS with proviral pNL4-3 DNA, immunoprecipitated these complexes using an anti-FLAG antibody and blotted for the presence of FLAG, UPF1 and UPF2 (Figure 7I). As expected, UPF1 co-immunoprecipitated with both UPF3aL and UPF3aS, but the UPF2 binding mutant, UPF3aS, did not pull down UPF2. Thus, it seems that UPF2, UPF3aL and UPF1 in complex are detrimental to HIV-1 expression by trapping the vRNA in the nucleus, while UPF3aS in complex with UPF1 does not have this effect due to the fact that it does not bind UPF2. Given that our results point to a negative regulation of vRNA export by UPF2, we decided to express wild type UPF2 (FLAG-UPF2) and a mutant of UPF2 which does not bind UPF1 (FLAG-UPF2^1–1096^, which has a deletion at the C-terminus) with proviral DNA pNL4-3 and assessed vRNA localization by FISH/IF co-analyses (Figure 7E,F). Wild type UPF2 blocked vRNA nuclear export (Figure 7E), similar to what was found for UPF3aL. The C-terminal mutant UPF2^1–1096^ exhibiting a complete loss of UPF1 binding failed to block vRNA export (Figure 7F). A point mutant of UPF2 (UPF2^1173^), which exhibits an incomplete association to UPF1, resulted in a clear 50% phenotype between nuclear and cytoplasmic localization of the vRNA ([73], see below). These results show that UPF2 blocks vRNA nucleocytoplasmic export by associating with UPF1.

Since the C-terminal domain of UPF2 binds the N-terminal CH-domain of UPF1 [48], we therefore decided to confirm that the deletion mutant UPF2^1–1096^ does not bind UPF1. Our results show that all of the UPF2 constructs immunoprecipitated with UPF3b but bound UPF1 differentially (Figure 7J). UPF1 immunoprecipitated with wild type UPF2 but weakly with, UPF2^1173^ explaining in part why there was only a partial 50% blockade to nuclear export of the vRNA (*n* > 50, in 3 independent experiments; [73]), despite a quantitative immunoprecipitation of FLAG-UPF2 proteins. On the other hand, UPF1 failed to immunoprecipitate with the C-terminal mutant of UPF2 (UPF2^1–1096^; Figure 7J) correlating with the loss in the ability to block the nuclear export of the vRNA. There was no significant change in vRNA levels under these conditions (Figure 7J, bottom). Importantly, it was shown that UPF2^1–1096^ was still able to bind UPF3aL and UPF3b despite its inability to bind UPF1 [48]. The characteristics and binding partners of UPF3 and UPF2 wild type and mutants used as well as a summary of their effects on Rev+ HIV-1 vRNA export are summarized in Table 2. These results demonstrate that the UPF3aL/UPF2-UPF1 association blocks vRNA nuclear export (Figure 7G) and that HIV-1 excludes UPF2 from its binding to UPF1 in order for UPF1 to promote HIV-1 vRNA export together with Rev, CRM1 and DDX3.

**Table 2:** UPF2 and UPF3 plasmids.

UPF Plasmids Used	Deleted Region	Protein Localization	Characteristics	Effect on HIV-1 RNA Export (Rev+ Conditions)	Interactions	
					UPF1	UPF2
UPF3b	NA	Nuclear and cytoplasmic	Forms complexes with UPF1, 2 and 3 aL [31,47,48]; regulates UPF3A levels [49]	No effect on Rev+ export [5] and this study	Yes	Yes
UPF3aL	NA	Nuclear and cytoplasmic	Forms complex with phospho-UPF1-UPF2 [31,47]; levels regulated by UPF3b, UPF2 [49]	Negatively affects Rev+ export (this study)	Yes	Yes
UPF3aS	Exon4 from UPF3aL residues 117–149	Nuclear and cytoplasmic	Exon 4 from UPF3aL [31]; forms complex with PUPF1-SMG5/7 [31]; does not bind UPF2 [31,48]	No effect on Rev+ export (this study)	Yes	No
UPF2^WT^	NA	Cytoplasmic*	Forms complex with UPF1, 3 aL, 3b [31,47,48,49]	Negatively affects Rev+ export (this study)	Yes	NA
UPF2^1–1096^	C-terminal deletion 1096 to 1272	Cytoplasmic	C-terminal deletion [48,53]; does not bind UPF1 [48,53]	No effect on Rev+ export (this study)	No	NA

NA: Not applicable; * Associates to nuclear proteins and may be resident in nucleus.

## 4. Discussion

In this study, we identify a novel function for UPF1 in regulating the HIV-1 vRNA fate. UPF1 appears to be acting at multiple levels of control during vRNA biogenesis. First, our results indicate that UPF1 homes the RRE-containing vRNA (Figure 1 and Figure 2). Second, considering that UPF1 overcomes nuclear HIV-1 vRNA retention in the absence of Rev (Figure 1), UPF1 thus represents a dominant adaptor protein that can associate to the vRNA in both Rev− and Rev+ conditions to promote nuclear export. This appears to be in the context of a nuclear HIV-1 export UPF1-containing RNP (Figure 4). Indeed, HIV-1 may have evolved the means to efficiently program the trafficking of its mRNAs by co-opting UPF1 function. Nevertheless, UPF1 only affects RRE-mediated, but not CTE-mediated export, similar to what was found for DDX3 [12]. The other similarity includes the observation that UPF1, as well as DDX3, bind *de* *novo* synthesized vRNA in the nucleus [44] and recent results show that UPF1 selectively associates to long 3'-UTR substrates [39], of which the vRNA is one. Interestingly, it was shown that UPF1 associates with transcription sites [84]. Not only does Rev cause the relocalization of CRM1 to transcription sites but Rev-CRM1-RRE-containing RNAs are released as one complex from these transcription sites [4]. Thus, it is likely that UPF1 is part of this complex since it was shown to be present at the transcription sites, associated with *de* *novo* synthesized HIV-1 vRNA and is part of a complex with Rev, CRM1, DDX3 and the HIV-1 vRNA.

Our results not only reveal that UPF1 is found in two different HIV-1 RNP complexes, one in which DDX3, CRM1, Nup62 and Rev are found, and another in which Gag is present (Figure 4), but also provide further support for both nuclear and cytoplasmic functions of UPF1 in HIV-1 replication. In a recent proteomic screen, UPF1 and UPF3b were found in association with Gag or Rev, amongst other viral proteins, strengthening the validity of our results [85] even though we have not observed UPF3b association with HIV-1 vRNA [73]. In the nucleus, UPF1 most likely enhances the association of the HIV-1 vRNA with export factors and then accompanies the vRNA into the cytoplasm where it enhances stability and translation independently of its nuclear remodelling function [5]. In addition, UPF1 has been found in virus particles [61,86] and purified viral cores [76] whose presence can be mediated by its interaction with the vRNA. Finally, our ongoing work using latently infected model cell systems reveals that when latent provirus is reactivated, a several-fold increase in UPF1 expression levels is the result, concomitant with the burst in Gag expression, while the levels of UPF2 and UPF3aL did not change significantly [73].

Our data suggest that nucleo-cytoplasmic shuttling of UPF1 requires the NES sequence present in the helicase, which would be expected to bind the NES-binding cleft in CRM1 competing with Rev for binding. However, recent studies by Frankel and colleagues showed that CRM1 forms a dimer within the Rev/RRE nuclear export complex [87] raising the possibility for the formation of a Rev/CRM1-CRM1/UPF1 complex. Moreover, based on our structural modeling and previous data, it is tempting to speculate that this viral nuclear export complex would be stabilized by Rev-UPF1, DDX3-UPF1 and DDX3-CRM1 [12] interactions. Indeed, a role for UPF1 in RNA export is not without precedence. Yeast-two hybrid studies have shown that UPF1 interacts with nucleoporins Nup100/116 (the mammalian homolog is Nup98), suggesting that this interaction ensures the association of UPF1 with the newly synthesized mRNAs [44,75,88]. Interestingly, Nup98 is also a cofactor of CRM1/Rev as well as an important nucleoporin involved in vRNA export [89]. Further studies revealed that Nup62 is a component of the Rev RNP [10] and recent work demonstrates that Nup62 is a host component involved in mediating vRNA nucleocytoplasmic export [15]. Ataxia-telangiectasia-mutated kinase (ATM), while involved in HIV-1 DNA integration and DNA damage, also promotes vRNA nucleocytoplasmic export by activating Rev [90] and ATM phosphorylates UPF1 [91]. Finally, recent work demonstrates co-transcriptional assembly of Rev, CRM1, and RRE-containing RNAs at the site of transcription, and now likely with the participation of UPF1, in one single export complex [4]. Future work will be needed to determine if the post-translational modification of UPF1 is required for its activity in nucleocytoplasmic export [90,92].

The second major finding is that UPF2-UPF3aL acts as a negative regulator of vRNA nuclear export. UPF2 was first found to be excluded from the HIV-1 RNP and mutants of UPF1 that do not bind UPF2 still upregulated vRNA translation [5]. We extended these findings to show that, whereas UPF2 expression blocks vRNA nucleocytoplasmic export, UPF2 mutants that do not bind UPF1 lose this ability (Figure 7). Moreover, our *in* *silico* analyses allowed us to gain insights on the molecular mechanism of the UPF2 exclusion, which is possibly mediated by the binding of Rev to the CH-domain of UPF1 at a site that overlaps with the UPF2 binding site (Figure 5). The regulatory circuit we described here also implicates UPF3a. Earlier work demonstrated that both isoforms of UPF3a (UPF3aL and UPF3aS) are in different complexes with UPF1 [31] termed the “post-phosphorylation complex” (UPF3aL-UPF2 with P-UPF1) and the “pre-dephosphorylation complex” (UPF3aS-SMG5/7-PP2A-P-UPF1) [31]. The presence of both of these complexes in the cell can explain why both UPF3a isoforms, which differ in their ability to bind UPF2, have differential effects on vRNA export. UPF3aL overexpression leads to the recruitment of both UPF2 and UPF1, blocking vRNA export, while UPF3aS only recruits UPF1 but excludes UPF2, resulting in little effect on nucleocytoplasmic vRNA export (Figure 7). The strict exclusion of UPF2 imposed by HIV-1 shown here and earlier [5] would also enhance UPF1’s ability to bind RNA, perhaps the vRNA in infected cells, by a conformational switch induced by this association [28] resulting in enhanced stabilization, nucleocytoplasmic export and translatability of the vRNA.

**Figure 8:**
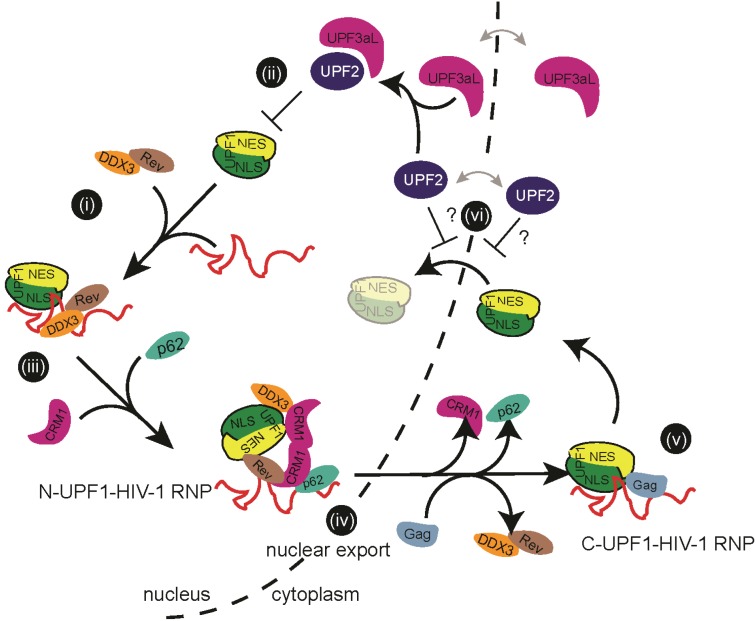
Proposed model for UPF1-mediated vRNA nucleocytoplasmic export. During the HIV-1 expression phase, the shuttling ability of UPF1 is important for vRNA nucleocytoplasmic export. In the nucleus, UPF1 can be recruited to the vRNA via its C-terminal domain (including the NLS domain, Figure 1A) and acts as an adaptor protein to bridge the nuclear HIV-1 RNP with export factors through, most likely, its NES region (**i**). However, UPF3aL can potentially block the recruitment of UPF1 to prevent the assembly of an export-competent nuclear RNP by recruiting UPF2 (**ii**). Once assembled, the UPF1 nuclear export complex, composed of the vRNA, UPF1, Rev, CRM1, DDX3 and DDX1 then associates with nucleoporins Nup98 and/or Nup214, as well as Nup62 to form a nuclear complex (*N*-UPF1-HIV-1 RNP, (**iii**)). This complex can then be efficiently exported (**iv**). Once in the cytoplasm, several factors including UPF1 recycle back to their original locations. UPF1 can either act in the context of a cytoplasmic RNP (C-UPF1-HIV-1 RNP) to promote vRNA stability and utilization (translation) to ensure Gag synthesis (**v**), or, according to the current results in this manuscript, escapes interaction with UPF2 (**vi**) within the cytoplasm or nucleus (“?” indicates open question) to control vRNA nucleocytoplasmic export.

Although the precise function of UPF2 in the fate of HIV-1 vRNA remains incompletely understood, its expression levels may directly influence the availability of UPF1. While UPF1 has been shown to shuttle between the nucleus and cytoplasm, UPF2 has been reported to be strictly cytoplasmic [21]. It is therefore conceivable that UPF2 prevents UPF1 from being imported to the nucleus, where it could enhance viral RNA production and export (see model presented in Figure 8). Nevertheless, several independent studies show that UPF2 localizes to nuclear fractions [31,93,94] and that UPF2 associates with UPF3aL (a nucleocytoplasmic shuttling protein), hinting at the possibility that UPF2 might also be a component of nuclear RNPs. Moreover, NMD and SMD are believed to be competitive mechanisms because UPF2 and Staufen1 bind to the same region in UPF1 (Figure 1A and [95]). Since UPF1 is a component of the Staufen1/HIV-1 RNP complex that excludes UPF2 [45], it is possible that HIV-1 mediates the association between UPF1 and Staufen1, occluding UPF2’s ability to associate to UPF1 [95]. Hence, we propose that high expression levels of UPF2 would lead to the formation of UPF1-containing cytoplasmic complexes and limit the availability of UPF1 to the nucleus, resulting in a block to HIV-1 vRNA export that depends on UPF1 nucleocytoplasmic shuttling.

The findings presented in this manuscript do not represent the first UPF2-independent function of UPF1 [25,41]. Our current model proposes that immediately following viral transcription, UPF1 is recruited to the vRNA via association to the NLS domain, the helicase IV-VI and/or C-terminal domains and to steer this intron-containing mRNA away from the nuclear RNA quality control and surveillance machinery (Figure 8, step (i)). UPF1 can then serve as an adaptor protein for Rev/CRM1-mediated vRNA export. The immediate recruitment of UPF1 is in-line with earlier [44] as well as recent findings on UPF1’s ability to bind long, 3'-UTRs [46] that precedes NMD [39] and on the early recruitment of Rev and CRM1 to sites of HIV-1 transcription [4]. The nuclear RNP (*N*-UPF1-HIV-1 RNP) will then acquire additional nuclear trafficking proteins including DDX3, DDX1, eIF5A, amongst others, that are important for vRNA nuclear export, and will position itself for nuclear export through the nuclear pore (Figure 8, steps (iii–iv)). The RNP engages several nuclear pore proteins, such as Nup98, Nup214 and/or Nup62, resulting in the efficient export and cytoplasmic localization of the vRNA. At this point neither the precise makeup of the viral nuclear export RNP is completely characterized nor do we have a full understanding of the direct and indirect intermolecular interactions that occur.

UPF2 does not play a functional role in several UPF1-containing mRNPs such as the UPF2-independent NMD RNP [41], the UPF1 RNP that sits on structured 3'-UTRs [96] and another that regulates histone mRNA stability during cell replication [25]. Our results suggest that UPF2 is excluded from HIV-1 RNPs through the selective or antagonistic interactions with viral and cellular proteins, such as Rev and/or Staufen1 that will invariably impact function. UPF2’s association to UPF1, for example, impacts UPF1 structure and influences enzymatic and other functions, so this scenario seems to be the most likely [28,53,97]. This might also be true for UPF1’s newly characterized role in HIV-1 reverse transcription of the vRNA early in HIV-1 infection, at which stage UPF2 still has no role [86]. Anti-viral strategies using the Rev-transdominant RevM10 to block vRNA nuclear export have failed because of the development of resistance, so targeting of Rev alone will likely not be sufficient. Therefore, cofactors such as DDX3 and UPF1 that assemble with and aid in Rev function remain viable targets [18]. Since UPF1 is required for vRNA stability and translation with an added functionality in the control of nucleocytoplasmic RNA export that implicates other UPF proteins, the targeting of this regulatory circuit could represent a suitable and multi-pronged approach that will contribute to the arsenal that we use to treat and eventually cure HIV-1 infection.

## 5. Conclusions

HIV-1 commandeers UPF host proteins for critical steps of the viral replication cycle. In this manuscript we demonstrate that HIV-1 ensures nuclear export of the genomic RNA by the recruitment of UPF1 but by the exclusion of UPF2.

## Supplementary Files

Supplementary File 1
