# Supplementary material for: HIV-1 Recruits UPF1 but Excludes UPF2 to Promote Nucleocytoplasmic Export of the Genomic RNA
Source: Biomolecules. 2015 Oct 20;5(4):2808–39. doi: 10.3390/biom5042808 (PMC4693258; doi:10.3390/biom5042808)
Supplement: Supplementary File 1 [file biomolecules-05-02808-s001.pdf]

Supplementary Materials

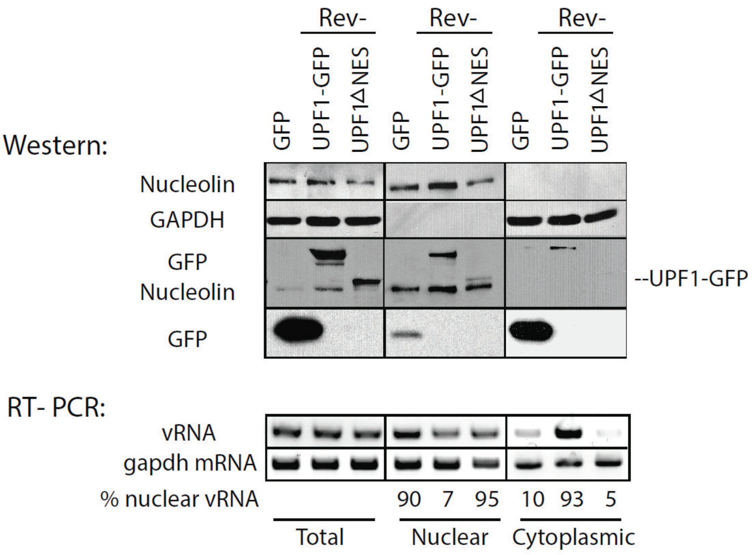

Figure S1. Wildtype UPF1 mediates the translocation of vRNA to the cytoplasm in Rev− conditions. Cells were transfected with empty vector, CMV-GFP, UPF1-GFP or UPF1 ΔNES with a proviral HIV-1 DNA harbouring a mutation to block Rev expression, as described in the manuscript. Nuclear and cytoplasmic fractions were prepared. Nucleolin and GAPDH were identified by Western blotting and served as nuclear and cytoplasmic marker proteins, respectively, to deminstrate the efficiency of fractionation procedure. vRNA and gapdh mRNA levels were identified by semi-quantitative RT-PCR. The numbers belo blots indicate vRNA abundance in nuclear and cytoplasmic fractions, and is representative of 2 experiments. Anti-GFP identified UPF1-GFP (wildtype UPF1 and UPF1 ΔNES) and GFP proteins. These results are related to and confer with data presented in Figure 1.

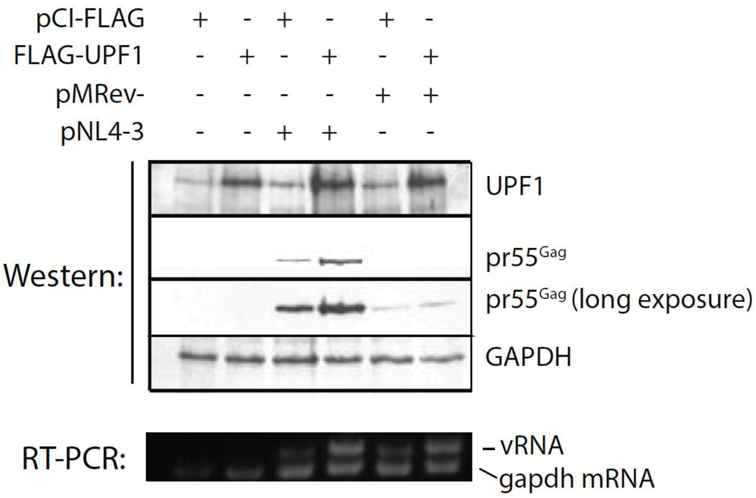

Figure S2. UPF1 overexpression increases HIV-1 Gag and vRNA expression levels in Rev+ and Rev- conditions. HeLa cells were either transfected just with pCI-FLAG or FLAG-UPF1WT or each co-transfected with either pNL4-3 (Rev+) or pMRev−. At 30 h post-transfection, cells were harvested for western blot analysis for UPF1, Gag (pr55Gag) and GAPDH (loading control). RNA was also isolated from cell extracts and RT-PCR was performed to estimate steady-state levels of vRNA and gapdh RNAs.

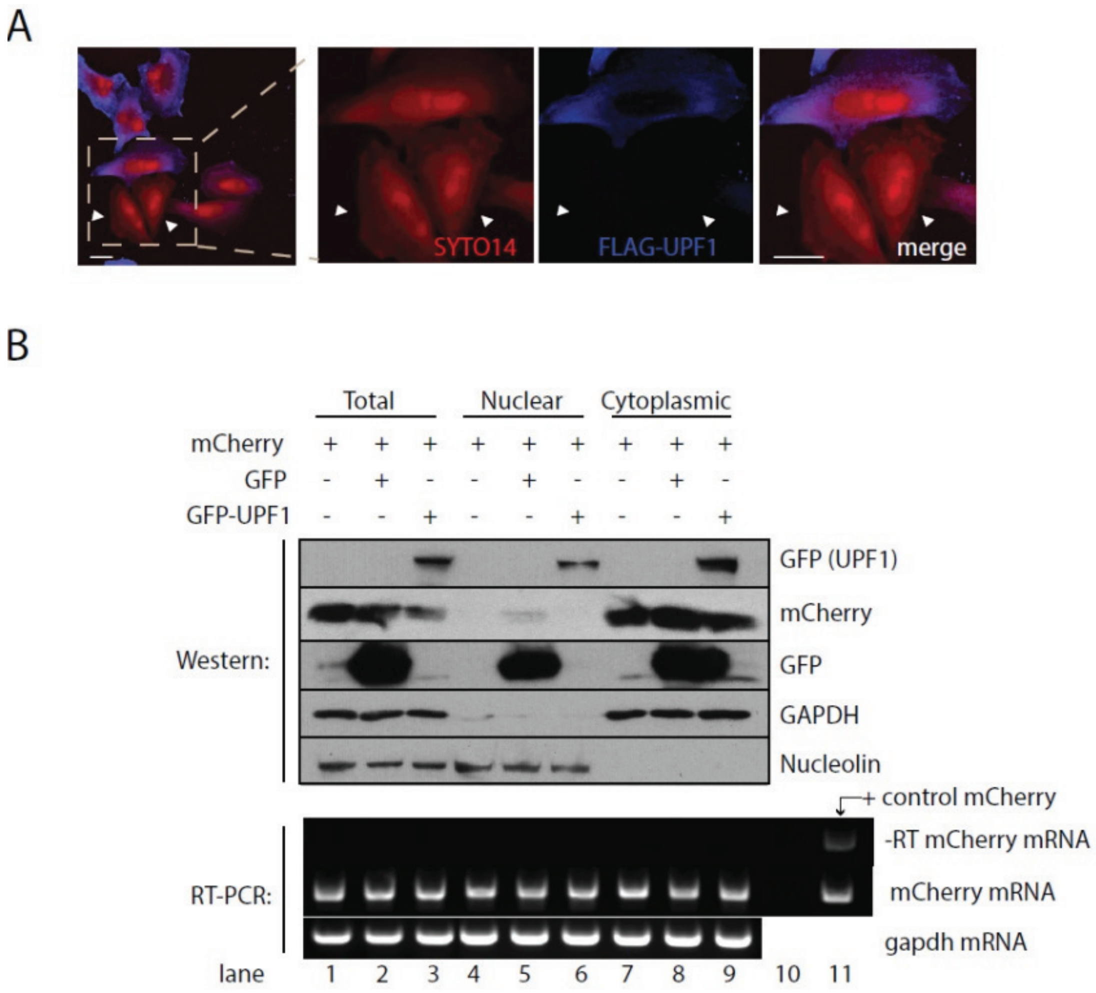

Figure S3. UPF1 overexpression does not affect the distribution of total cellular RNA or that of mCherry protein and mRNA. (**A**) HeLa cells were seeded onto coverslips and transfected with FLAG-UPF1. At 30 h post-transfection, cells were fixed for immunofluorescence analysis for FLAG (UPF1, blue) and total cellular mRNA was stained with SYTO14 (red). Wjite arrowheads indentify non-transfected cells. Size bars, 10 µm. The RNA localization shown in the images is representative for >90% cells (n = 60) from 2 experiments; (**B**) HeLa cells were mock transfected with mCherry (lanes 1, 4 & 7) or co-transfected with mCherry and GFP (lanes 2, 5 & 8) or GFP-UPF1 (lanes 3, 6 & 9). At 30h post-transfection, cells were harvested and fractionation was carried out as described in Materials and Methods. Western blot analysis was performed for GFP, mCherry, nucleolin (nuclear compartment marker) and GAPDH (cytoplasmic compartment marker). RNA was also isolated from cell extracts and cDNA followed by PCR, performed to measure levels of mCherry (−RT followed by +RT) and gapdh mRNA. mCherry mRNA signals, relative to those for gapdh range from 1.0 (arbitrarily set, mCherry alone) and vary around 1 ± 0.04. The last lane represents the positive control for the PCR reaction with the mCherry expression plasmid. These results are related to and confer with data presented in Figure 1.

© 2015 by the authors; licensee MDPI, Basel, Switzerland. This article is an open access article distributed under the terms and conditions of the Creative Commons Attribution license (http://creativecommons.org/licenses/by/4.0/).
